# Placenta Accreta Spectrum (PAS) disorders: incidence, risk factors and outcomes of different management strategies in a tertiary referral hospital in Minia, Egypt: a prospective study

**DOI:** 10.1186/s12884-019-2466-5

**Published:** 2019-08-27

**Authors:** Saad El Gelany, Mohammed H. Mosbeh, Emad M. Ibrahim, Mo’men Mohammed, Eissa M. Khalifa, Ahmed K. Abdelhakium, Ayman M. Yousef, Heba Hassan, Khaled Goma, Ahmed Abd Alghany, Hashem Fares Mohammed, Ahmed M. Azmy, Wegdan A. Ali, Ahmed R. Abdelraheim

**Affiliations:** 1 Obstetrics and Gynaecology Department, Faculty of Medicine, Minia Maternity and Children University Hospital, Minia University, Minia, Egypt; 2 Department of Anaesthesia and Intensive care, Faculty of Medicine, Minia University, Minia, Egypt

**Keywords:** Placenta accreta spectrum, Cervix, Tamponade

## Abstract

**Background:**

Placenta accreta spectrum (PAS) disorders have become a significant life-threatening issue due to its increased incidence, morbidity and mortality. Several studies have tried to identify the risk factors for PAS disorders. The ideal management for PAS disorders is a matter of debate. The study objectives were to evaluate the incidence and risk factors of PAS disorders and to compare different management strategies at a tertiary referral hospital, Minia, Egypt.

**Methods:**

This prospective study included 102 women diagnosed with PAS disorders admitted to Minia Maternity university hospital, Egypt between January 2017 to August 2018. These cases were categorized into three groups according to the used approach for management: *Group (A)*, (*n* = 38) underwent cesarean hysterectomy, *group (B)*, (*n* = 48) underwent cesarean section (CS) with cervical inversion and ligation of both uterine arteries and *group (C)*, (*n* = 16): the placenta was left in place.

**Results:**

The incidence of PAS disorders during the study period was 9 / 1000 maternities (0.91%). The mean age of cases was 32.4 ± 4.2 years, 60% of them had a parity ≥3 and 82% of them had ≥2 previous CSs. Also, 1/3 of them had previous history of placenta previa. Estimated blood loss (EBL) and blood transfusion in group A were significantly higher than other groups. Group (C) had higher mean hospital stay duration. Group A was associated with significantly higher complication rate.

**Conclusions:**

The incidence of PAS disorders was 0.91%. Maternal age > 32 years, previous C.S. (≥ 2), multiparity (≥ 3) and previous history of placenta previa were risk factors. The management of PAS disorders should be individualized. Women with PAS disorders who completed their family should be offered cesarean hysterectomy. Using the cervix as a tamponade combined with bilateral uterine artery ligation appears to be a safe alternative to hysterectomy in patients with focal placenta accreta and low parity desiring future fertility. Patients with diffuse placenta accreta keen to preserve the uterus could be offered the option of leaving the placenta aiming at conservative management after proper counseling.

**Trial registration:**

Registered 28th October 2015, ClinicalTrials.gov NCT02590484.

## Background

Placenta accreta spectrum (PAS) disorders have become a significant life-threatening obstetrical issue due to its increased incidence from 0.12 to 0.31% in the last 30 years and the reported mortality rate of approximately 7.0% [1]. In addition, it is related to considerable maternal morbidity which includes massive blood transfusion, urinary tract injury, hysterectomy, admission to intensive care unit (ICU) admission, sepsis, and long hospital stay [2]. The term PAS refers to variable degrees of adherence and invasion of the uterus and / or surrounding organs by the placenta, i.e. placenta accreta, increta and percreta [3] which obstruct the placental separation at delivery and could consequently result in considerable maternal hemorrhage that menace the life of both the mother and the neonate [4]. Recently, several studies have tried to identify the risk factors for PAS disorders, it has been reported that maternal age (≥ 35 years) and placenta previa were significantly associated with the development of PAS disorders [5]. Likewise, advanced maternal age and increased number of previous CSs were independent risk factors for PAS disorders [6], however, Zhang et al., (2017) added parity as another risk factor [1].

The ideal management approach for PAS disorders is controversial [7]. The American College of Obstetricians and Gynaecologists (ACOG) recommends elective CS hysterectomy with the placenta in place as removal of the placenta in these cases leads to massive blood loss [8]. This option may be not accepted in cases wishing to preserve their fertility. In such cases, conservative management should be considered after proper counseling regarding risks [7].

Conservative management of PAS disorders involves all techniques that aim to preserve the uterus. It includes piecemeal removal of the placenta (the extirpative technique); the expectant management through leaving the placenta; the Triple-P procedure; and many other conservative surgical techniques. These methods have been used alone or in combination to reduce haemorrhage associated with PAS disorders [3].

The expectant management aims to decrease severe maternal morbidity during CS for PAS disorders [9–12]. Forcible manual removal of the placenta [13]—increases the risks of severe haemorrhage, hysterectomy, coagulopathy and injuries to surrounding organs [9–12].

The objectives of this study were to evaluate the incidence and possible risk factors of PAS disorders and to compare different management strategies regarding their outcomes at Minia Maternity and Children university hospital, Egypt.

## Methods

### Setting

Minia Maternity and Children university hospital, Egypt between January 2017 to August 2018.

### Ethical considerations

The study has been approved by the research ethics committee of the department of Obstetrics and Gynaecology, Faculty of Medicine, Minia University. All patients had signed a written informed consent after they have been made aware of the purpose of the study, interventions, outcome and possible complications.

### Study participants

#### Inclusion criteria

- All patients diagnosed prenatally as PAS disorders by means of Ultrasound, Doppler, and magnetic resonance imaging (MRI).

#### Exclusion criteria

- Impaired liver or renal functions
- Coagulation disorders.
- Those with spontaneous separation of placenta intraoperative or any other associated uterine pathology needing hysterectomy.
- Patient refusal to participate in the study.

### Study plan

In this study, we followed 102 patients attended to our hospital and were diagnosed with PAS disorders antenatally and scheduled for different management modalities. History taking, general, abdominal examinations, laboratory and radiological investigations were done for all participants. Four units of cross-matched blood were booked. Histopathological examination was performed for all cases underwent cesarean hysterectomy.

### Operative interventions

After delivery of the fetus, gentle cord traction was applied to see if the placenta will separate or not unless percreta is confirmed intraoperatively. The following clinical scenarios and management modalities were seen in our cases:

(A). In cases with spontaneous partial placental separation, the remaining portion is removed and bleeding is dealt with by cervical tamponade and bilateral uterine artery ligation was performed [14, 15]. Cesarean hysterectomy was performed if the bleeding persisted.
(B). In cases of complete invasion (diffuse accreta or percreta) either hysterectomy or leaving the placenta in situ followed by removal later on were performed according to the parity of the patient and her wishes to preserve the uterus or not.

Then patients were admitted to post-operative ward or ICU.

All patients in group C have been properly counselled and informed that they might need re-exploratory surgery to remove the placenta later on. Four patients (out of 20 patients) declined this and offered the other option which was cesarean hysterectomy. Additionally in Group C, Ultrasound, Doppler and MRI examinations were done for measuring the placental volume, vasculature, and size of uterus. In group C, postoperative embolization of uterine arteries (UAE) was done in cases with placenta percreta and /or extensive placental vascularity. A dedicated team experienced in managing cases of PAS including the authors list decide the optimal time of intervention to remove the placenta depending on the clinical, ultrasound, Doppler and MRI features.

Outcome measures include successful management plan, maternal mortality and morbidity (admission to ICU, massive blood transfusion, coagulopathy, bladder injury, infection and hospital re-admission within 6 weeks).

### Statistical analysis

SPSS program (Statistical Package for Social Sciences, version 20, IBM, NY, USA) was used for statistical analysis. Mean ± standard deviation (SD) and range were calculated for numerical data, while number and percentage were calculated for categorical data. For comparisons of quantitative data, we used independent, paired sample T-test and One way ANOVA test. For comparisons of qualitative data, Chi-square test or Fisher exact was used. Probability values (P. V.) were considered significant if less than 0.05 and highly significant if less than 0.01.

## Results

The present study included a total of 102 cases diagnosed as PAS disorders with an incidence of 0.91% during the study period. These cases underwent different modalities for management of PAS disorders and were categorized into three groups as follow:

- Group (A), (*n* = 38, 37.3%): underwent cesarean hysterectomy.
- Group (B), (*n* = 48, 47.0%): underwent CS with cervical inversion and bilateral ligation of both uterine arteries.
- Group (C), (*n* = 16, 15.7%): Leaving placenta in place.

Group A represent the radical management group in which cesarean hysterectomy was performed (38/102 cases; 37.3%) while Groups (B and C) represent the conservative management groups; cases of PAS disorders managed without hysterectomy with preservation of the uterus (64/102 cases; 62.7%).

Table 1 presents the baseline characteristics of all studied cases.

**Table 1:** Baseline characteristics of studied group

Variable		Descriptive (*n* = 102)
Age, mean ± SD (range)		32.4 ± 4.2 (23–39)
Parity	1–2	42 (41.2%)
	3–4	46 (45.1%)
	≥ 5	14 (13.7%)
Previous CS	1	18 (17.6%)
	2	30 (29.4%)
	3	36 (35.3%)
	4	18 (17.6%)
History of placenta previa	Yes	34 (33.3%)
	No	68 (66.7%)

*SD* standard deviation, *CS* cesarean section

Comparisons of baseline characteristics among the three groups is shown in Table 2.

**Table 2:** Comparisons among the three groups regarding baseline characteristics

Variable		Group A (*n* = 38)	Group B (*n* = 48)	Group C (*n* = 16)	P. V. (Sig.)
Age, mean ± SD (range)		32.9 ± 4.1	31.8 ± 4.2	31.7 ± 4.5	0.429^NS^
Parity	1–2	2 (5.3%)	26 (54.2%)	14 (87.5%)	< 0.001**
	3–4	22 (57.9%)	22 (45.8%)	2 (12.5%)	
	≥ 5	14 (36.8%)	0	0	
Previous CS	1	0	10 (20.8%)	8 (50%)	< 0.001**
	2	4 (10.5%)	18 (37.5%)	8 (50%)	
	3	18 (47.4%)	18 (37.5%)	0	
	4	16 (42.1%)	2 (4.2%)	0	
History of placenta previa	Yes	13 (34.2%)	15 (31.2%)	6 (37.5%)	0.035*
	No	25 (65.8%)	33 (68.8%)	10 (62.5%)	

*NS* Not significant, * = Significant (*p* < 0.05),** = highly significant (*p* < 0.01), *SD* standard deviation, *CS* cesarean section

Table 3 demonstrates Comparisons among the three groups regarding clinical, laboratory findings and postoperative morbidities.

**Table 3:** Comparisons among the three groups regarding clinical, laboratory findings and postoperative morbidities

Variable			Groups			P. V. (Sig.)
			Group (A) (*n* = 38)	Group (B) (*n* = 48)	Group (C) (*n* = 16)	
Pre op. Hb (g/dl)			11.3 ± 0.3	11.4 ± 0.7	11.4 ± 0.4	0.665^NS^
Post op. Hb (g/dl)			9.5 ± 0.4	9.7 ± 0.5	10.7 ± 0.5	0.047*
Decrease in Hb (%)			15.7 ± 3.8	14.9 ± 4.1	8.6 ± 2.7	0.036*
Estimated blood loss (L)			2.84^a^ ± 1.12	2.58 ^**b**^ ± 1.03	2.12 ^**c**^ ± 0.87	**0.048**
Blood transfusion (units)			3.8^**a**^ ± 1.2	3.7 ^**a**^ ± 1.1	2.9 ^**b**^ ± 0.6	0.018*
Mean duration of hospital stay (days)			6.8 ^**b**^ ± 1.8	5.1^**c**^ ± 1.8	8.4 ^**a**^ ± 1.4	< 0.00**
Hospital stay (day)	2		2 (5.3%)	4 (8.3%)	0	< 0.00**
	3–5		4 (10.5%)	30 (62.5%)	0	
	6–7		14 (36.8%)	6 (12.5%)	8 (50%)	
	> 7		18 (47.4%)	8 (16.7%)	8 (50%)	
CCComplications	No		10 (26.3%)	32 (66.7%)	9 (56.25%)	< 0.00**
	Yes	Bladder injury	15 (39.5%)	4 (8.3%)	1 (6.25%)	
		1ry PPH	0	6 (12.5%)^d^	0	
		2ndry PPH	0	0	2 (12.5%)	
		ICU admission	8 (21.1%)	4 (8.3%)	1 (6.25%)	
		Coagulopathy	4 (10.5%)	2 (4.2%)	0	
		Infection	0	0	2 (12.5%)	
		Delayed hysterectomy	0	0	2 (12.5%)	
		Re-exploration	1 (2.6%)	0	0	
Mortality			0	0	0	0

*NS* Not significant, * = Significant (*p* < 0.05), ** = highly significant (*p* < 0.01), *HB* Haemoglobin, *PPH* Postpartum haemorrhage, *ICU* Intensive care unit, a, b, c,Means with different superscript in the same raw are significantly different

^d^in group B, six cases developed primary PPH after the primary procedure (cervical tamponade), four of them had hysterectomy and the remaining 2 cases were managed conservatively by insertion of intrauterine Bakri balloon

The total number of cases in group A was 38 cases. Thirty-one cases were placenta previa totalis and 7 cases were percreta without previa totalis.

In group C, 10 cases had postoperative uterine artery embolization. The mean duration of leaving the placenta in place was 54.4 ± 11.7 days (range 32–72 days). All cases in which the placenta left in place underwent elective laparotomy later on to remove the placenta. Vaginal route was tried in three cases but was not successful and conversion to laparotomy needed. The placenta was removed successfully in 14 cases out of 16 (87.5%) while two cases had delayed hysterectomy.

## Discussion

Both maternal and fetal morbidity and mortality from PAS disorders represent a major challenge to the obstetricians. The incidence of PAS disorders in the present study was 9 / 1000 maternities overall (0.91%), this incidence is comparable with published studies in the last decade (0.4–0.9%) [16]. But, our obtained incidence is higher than that of a recent study conducted in another university hospital in Egypt for 1 year (2015) which reported that the incidence of PAS disorders was 0.33% [17]. Also, an incidence of 1 in 533 deliveries (0.2%) was reported earlier between 1982 and 2002 [18]. Recently, Carusi (2018) reported that the exact incidence of PAS disorders is not easy to ascertain, but it is about 1/1000 deliveries and this incidence is increasing along with increasing the risk factors [19]. This upsetting increase of PAS disorders could be explained by the increasing rates of caesarean sections plus increasing maternal age at delivery as it has been reported by many authors [16, 18–20]*.* Also, another possible explanation for the higher obtained incidence in the present study is that the studied hospital is the main tertiary referral hospital in our governorate which is populated by more than 5 million people.

The present results revealed that risk factors for PAS disorders were maternal age, > 32 years, previous cesarean sections (≥ 2), multiparity (≥ 3) and previous history of placenta previa. These results agreed with many authors, Fitzpatrick et al. studied risk factors for PAS disorders and found that high maternal age, prior caesarean delivery and placenta previa were considered as significant risk factors [21]. Also, another study in 2017 reported that older maternal age, prior caesarean section, placenta previa and high parity were independent risk factors for PAS disorders [22]. Also, other investigators reported similar results [1, 23].

In the current study, the results showed no significant differences among groups in mean age. However, group A (cesarean hysterectomy group) had significantly higher parity, followed by group (B) then group (C), (*p* < 0.01) and exactly the same trend of these results was found in previous cesarean sections (89.5% of cases in group A and 41.7% of cases in group B had ≥3 previous CSs compared to none cases in group C, this difference is highly significant, *p* < 0.01). These results agreed with kayem et al. [9].

The present results revealed that the majority of PAS disorders cases needed blood transfusion. This result was supported by a recent study in 2018 reported that 94.7% of cases with PAS disorders received blood transfusion [17], also another study reported that about 75.0% of PAS disorders cases needed blood transfusion [24]. So, blood transfusion should be anticipated in these cases, in addition, some cases may need massive transfusion.

In our study, group (A) had significantly higher estimated blood loss and blood transfusion compared to groups B& C (conservative management). The results of more bleeding and more need for blood transfusion in group A in our study could be explained by the nature of cases as most of cases managed by this modality was diffuse placenta accreta or placenta previa totalis percreta with massive hemorrhage so cesarean hysterectomy was performed. Additionally, the placental tissues could be interrupted unintentionally during surgery but our protocol was cesarean hysterectomy with the placenta in place if feasible. Also, in the cesarean hysterectomy group, downward displacement of the bladder is associated with bleeding from varicosities on the surface of the bladder and in the vesico-uterine pouch. Many studies in the literature support this finding [25–29]. High blood loss is the main drawback of cesarean hysterectomy done for PAS disorders [25]. Wright et al. (2011) reported that the mean blood loss for PAS disorders cases undergoing cesarean hysterectomy was 3000 ml, whereas the mean required packed red blood cell (PRBC) units for transfusion was 5 units. An estimated blood loss of ≥5000 mL was found in about 41.7% of women with a known diagnosis of PAS disorders [26]. Our results are also in agreement with the results with of Epstein et al. who conducted a study on 77 women with PAS disorders. There was a statistically significant higher EBL in the hysterectomy group in comparison to the conservative management group (2989 ml vs. 1410 ml) [27]. Our results are also in agreement with other studies in the literature reporting that conservative management led to reduce the cases which need blood transfusion than extirpative management do [9, 28, 29]. A retrospective study comparing expectant management versus extirpative management in two successive periods reported a reduction in blood transfusion, DIC, hysterectomies, and sepsis during the second period of conservative management in comparison with extirpative management [30].

The outcome of expectant management of PAS disorders was evaluated by a large French multicenter retrospective study. The uterus was preserved in 78% of cases compared to 87.5% in group C in our study. Overall, these data suggest that conservative management may be convenient in cases wishing to conceive further with agreement for follow up [10].

Additional procedures such as embolization, pelvic devascularization (permanent or temporary) have been used in conjunction with a conservative approach to speed up placental absorption. Some authors also reported that these procedures prevent the occurrence of secondary postpartum haemorrhage [31, 32]. In group C of our study, uterine artery embolization (UAE) was performed in 10 cases out of 16 patients in whom the placenta was left in place. We also observed that UAE helped to decrease the placental vascularity and accelerated placental resorption and this is in agreement with earlier studies [31, 32].

In our study, complications were statistically significantly in hysterectomy group (73.7%) compared to other management modalities. Bladder injury was recorded in 39.5%. Our findings were supported by many studies which reported that complications after cesarean hysterectomy were higher with bladder and ureteric injuries are the most common injuries reported [26, 33–36].

Our results demonstrated that the mean hospital stay duration in CS hysterectomy group was 6.8 days. Similar to our findings, it has been reported that mean hospital stay ranged from 4 to 8 days after CS hysterectomy [26, 37].

In our study, the success rate of conservative management (group B and C) was 90.6%. Many authors reported that PAS disorders management conservatively is associated with reduction of hysterectomy and consequently fertility preservation [38–40]*.*

In addition to expectant management to preserve the uterus, there are many alternative conservative surgical techniques for stopping severe blood loss associated with PAS disorders, including balloon insertion [41], the B-Lynch maneuvers [42], compression sutures [43], a square sutures [44] and hypogastric (internal iliac) artery ligation [45], however the success of these techniques is variable [46, 47].

A novel conservative approach for PAS disorders called the “Triple-P procedure” was introduced [12, 40]. A reduction in the rates of postpartum haemorrhage and hysterectomy was reported after introduction of the Triple-P procedure [12]. However, this needs to be demonstrated in larger studies [3].

Our team has published two studies about conservative management of PAS disorders [14, 15]. The 1st study included 40 cases in which the cervix was used as a natural tamponade to control bleeding associated with placenta previa accreta. Hysterectomy was avoided in 38 out of 40 patients [14]. The 2nd study compared three different intraoperative techniques to reduce bleeding associated with PAS disorders. It was found that cervical inversion and ligation of both uterine arteries ligation are successful in reducing haemorrhage associated with PAS disorders [15]. This technique was used in cases with partial placental separation (focal accreta). This is in agreement with the FIGO consensus guidelines on placenta accreta spectrum disorders which stated that conservative management of PAS disorders could be used in cases with spontaneous partial separation if the invasive part of the placenta is not penetrating deeply into the uterine wall or laterally into the broad ligament [48].

Subsequent fertility and outcome of pregnancies following successful conservative management of PAS disorders do not appear to be affected. The major risk that the women should be informed about is the high risk of recurrence of PAS which may reach 28% [49].

It has been reported that the mortality rate of PAS disorders was approximately 7.0% [1]. Similarly, a recent study in Egypt found that mortality rate in PA cases was 3.2% [17]. However, in a nationwide study in USA, a mortality rate of 1.0% was reported in women who underwent obstetric hysterectomy [25], whereas other studies have described mortality rates of 1–6% [50–52]. Fortunately, we did not report any mortality in the current study.

The study limitations were the shortness of studied period, absence of long term follow up data and that we did not include perinatal outcome. Although the authors think it is difficult to do a randomized study in cases of PAS disorders as we select the best suitable option for each case. However, this is considered one of the limitations of the study.

## Conclusions

The incidence of PAS disorders in the current study was 9 / 1000 maternities (0.91%) which is slightly higher than other reported studies. Maternal age > 32 years, previous C.S. (≥ 2), multiparity (≥ 3) and previous history of placenta previa were risk factors for PAS disorders. The management of PAS disorders should be individualized. Women with PAS disorders who completed their family should be offered cesarean hysterectomy. Using the cervix as a tamponade combined with bilateral uterine artery ligation appears to be a safe alternative to hysterectomy in patients with focal placenta accreta and low parity desiring future fertility. Patients with diffuse placenta accreta keen to preserve the uterus could be offered the option of leaving the placenta aiming at conservative management after proper counseling.

## Data Availability

The datasets used and/or analyzed during the current study are available from the corresponding author on reasonable request.

## Abbreviations

ACOG: American College of Obstetricians and Gynaecologists
C.S.: Cesarean sections
EBL: Estimated blood loss
ICU: Intensive Care Unit
MRI: Magnetic Resonance Imaging
PAS: PAS disorders spectrum
PRBC: Packed Red blood Cell
PV: Probability Value
SD: Standard Deviation
UAE: Uterine Artery Embolization
